# A review of key technologies on flower picking robot: from perception, planning to non-destructive operations

**DOI:** 10.3389/fpls.2026.1945189

**Published:** 2026-09-03

**Authors:** Zhe Du, Pengyu Yi, Xin Jin, Chunyan Liu, Xinping Li, Zhitao He, Xiaolin Xie

**Affiliations:** College of Agricultural Equipment Engineering, Henan University of Science and Technology, Luoyang, China

**Keywords:** end effector, flower plant, picking robot, robotic arm, visual system

## Abstract

Flower picking is a labor intensive process heavily in the floriculture industry, and it remains predominantly manual. With the increasing shortage of agricultural labor and the continuous rise in labor costs, the sustainable development of the flower industry is facing severe challenges. Therefore, the using of robotic systems to picking flower plants has become an inevitable trend in the development of modern agriculture. This paper presents a systematic review of recent advances in flower picking robot, with a particular focus on system architecture and core enabling technologies. The core technologies are visual perception and target recognition, motion control and path planning, and flexible, non-destructive end effector design. The research status and limitations of these key technologies are analyzed, with special attention to their applications in both open field and controlled facility environments. Current challenges include low recognition accuracy under complex lighting and occlusion, insufficient end effector adaptability to diverse varieties, and low overall picking efficiency. Future directions include multiple modal sensing integrated with artificial intelligence, bionic and soft robotic end effectors, and multiple robot collaborative system. Finally, based on the perspectives of design and application, the review provides future research directions toward industrialization, with a particular focus on key enabling technologies of these technologies.

## 1 Introduction

China is the world’s largest producer of flower, with a planting area of approximately 1.4 million hectares and an annual output value of about 520 billion yuan (People's Daily Online, 2025). In 2024, China’s total import and export trade volume of flowers reached 782 million US dollars, a 10% rise from a year earlier. Potted plants, fresh cut flowers, and fresh cut branches (leaves) have a good market demand. With the continuous expansion of the scale of China’s flower industry, the traditional manual picking model is facing severe challenges (Wang et al., 2022). In addition to the continuous rise in labor costs, manual picking also faces problems such as low efficiency, inconsistent picking standards, and a high rate of flower damage (Liu and Liu, 2024). Moreover, with the aging of the population and urbanization, the large scale development of the flower industry has been severely restricted. With the development of agricultural modernization and precision agriculture technology, flower picking robotics technology has emerged, providing a feasible and efficient solution to the labor intensive challenges in the harvesting process of high value flowers (Choi et al., 2015). In comparison with manual picking, the temporal and spatial constraints of human labor can be overcome by robotic systems to realize uninterrupted, high efficiency harvesting (Zhang et al., 2020c). Meanwhile, post harvest damage rates can be minimized via precise force control and visual guidance technologies, with the marketability and competitive edge of floral products consequently enhanced to a notable extent (Feng et al., 2021).

**Figure 1:**
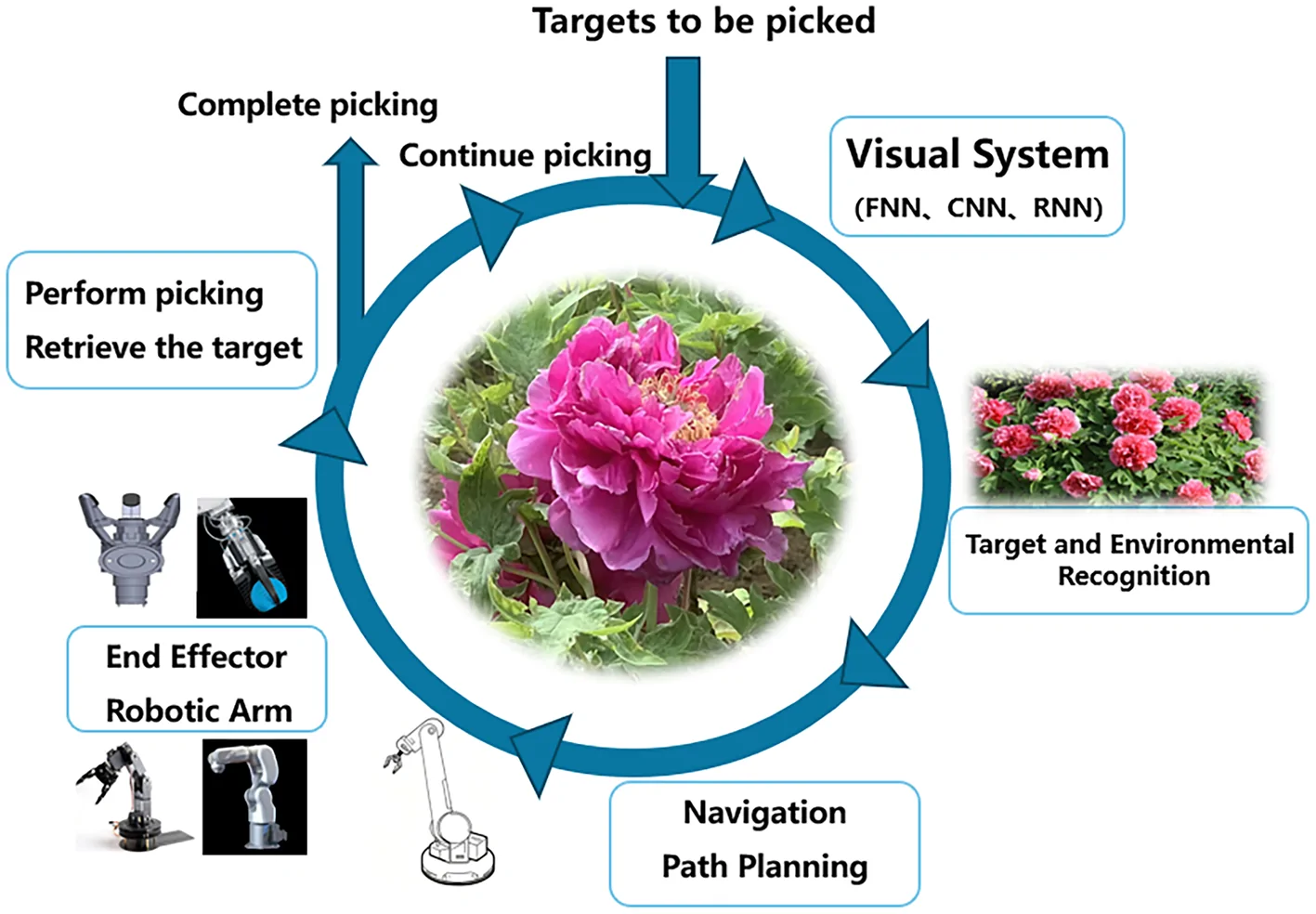
Basic concepts of flower picking robot.

Currently, the research and application of picking robots are mainly concentrated in the field of fruits, vegetables and crops, with some products having entered the commercial demonstration stage. In contrast, the research and application of flower picking robots are relatively limited. This situation of more in fruits and vegetables, less in flowers is primarily attributed to the significant differences between fruits, vegetables and flowers in terms of the characteristics of the picking objects. In greenhouses or open field environments, flowers are usually densely planted with interlaced branches and leaves that shield each other (Zhao et al., 2012), and lighting conditions are highly variable, posing enormous challenges to the stable recognition of visual systems (Torre et al., 2024). A wide variety of flowers exists with diverse shapes, colors, sizes, and textures. Their stems are slender, and petals are tender, which are extremely prone to bruising and crushing during mechanical grasping (Bac et al., 2014). As noted by Ni et al. (2018) and Tao et al. (2025), extremely high compliance and precision are therefore required for the end effector. The picking robot must accurately judge flower maturity, plan a collision free picking trajectory (Hua et al., 2025), precisely locate the cutting or grasping point on the pedicel within complex flower clusters, and complete a gentle separation operation. The entire operational workflow involves the close coordination of multiple subsystems, including target perception and recognition, motion control and path planning, and end effector design and compliant grasping (Duan et al., 2021; Yajun et al., 2022). Hongyu et al. (2022) further pointed out that the compliant grasping capability of the end effector directly affects the picking success rate. Gongal et al. (2015) emphasized the synergistic relationship between visual localization and motion control. A failure at any stage can lead to picking failure or flower damage, making the development of flower picking robots far more difficult than that of general fruit, vegetable, and crop harvesting robots. The overall operational workflow of a flower picking robot is illustrated in **Figure 1**.

In recent years, remarkable progress has been made in picking robots in terms of visual system recognition accuracy, robotic arm structure optimization, end effector improvement, and mobile platform upgrading, laying a solid foundation for the design, optimization, and application of flower picking robots. Numerous challenges remain to be urgently addressed. Recognition accuracy under complex lighting and occlusion conditions needs improvement. The adaptability of end effectors to diverse flower varieties is insufficient. Overall picking efficiency remains low. And the cost of core components is excessively high (Wang et al., 2020). More importantly, existing research mostly focuses on the improvement of individual technologies, while systematic review work that examines the coupled perception, decision and execution chain in flower harvesting from a system level perspective is still lacking. Most existing reviews are oriented toward general fruit, vegetable and crop robots (Zhang et al., 2020a; Chen et al., 2024a), or focus only on the technological progress of a single subsystem, failing to systematically identify the core bottlenecks and research gaps specific to the biological characteristics and operational environments of flowers.

Despite the aforementioned challenges, several representative advances have been made in flower picking robotics. For honeysuckle harvesting, the AI driven robot developed by China Agricultural University adopted an improved YOLOv8-pose algorithm for bud posture estimation and an integrated rotary gripping suction end effector. For fresh cut flowers, a demonstration in Yunnan showcased a robot system focusing on recognition accuracy, shearing efficiency, and terrain adaptability, marking a crucial step toward the integration of agronomy, agricultural machinery, and facility cultivation. These cases, though limited in number, demonstrate the feasibility and potential of robotic solutions for flower picking.

Based on the above analysis, this paper aims to provide a systematic review of recent advances in flower picking robots, with particular emphasis on their system architecture and core enabling technologies. This review systematically examines the current research status and existing problems in environmental perception and target recognition, motion control and path planning, and compliant non-destructive end effector design. It also compares and analyzes the differentiated technical requirements of flower picking robots in open field and controlled facility environments. Furthermore, in response to the existing challenges, it proposes innovative future research directions. This review provides a comprehensive reference for the design, optimization, and application of flower picking robots, and offers new insights for future research in this field.

## 2 Evolution and progress of flower picking robot

### 2.1 Research on the development history of flower picking robot

**Figure 2:**
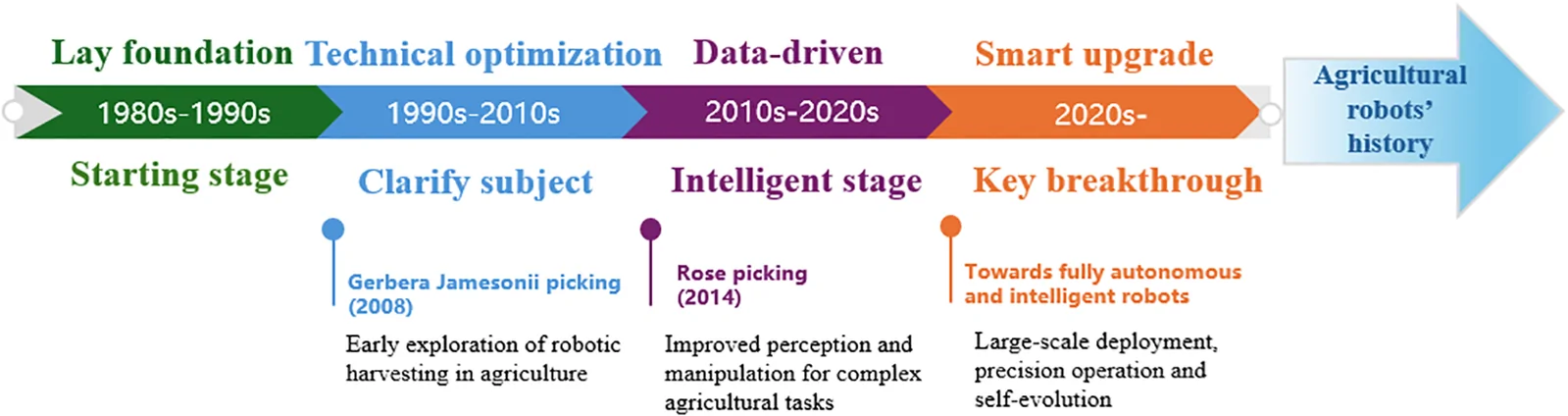
Agricultural picking robots’ history.

Based on advanced industrial foundations and artificial intelligence technologies, international picking robot have formed a relatively mature technical system, especially excelling in high precision flower picking operations in structured environments. Research and application of flower picking robot in China started relatively late. The individual technologies of the visual recognition and mechanical arm control have made significant breakthroughs, and the number of demonstration application cases had certain breakthroughs. However, there are still obvious gaps compared with the international advanced level in terms of overall system reliability, environmental adaptability, cost control, and large scale application level, as illustrated in **Figure 2**.

#### 2.1.1 Development of the international flower picking robot

In the 1970s, the exploration of agricultural robot applications was initiated in foreign countries. The first tomato picking robot was developed by researchers from Kyoto University (Kawamura et al., 1985), which also marked the inception of research on flower picking robots in 1985. Throughout the 1990s, substantial advancements were achieved in overseas picking robot technology. Robots capable of picking various fruits, including cucumbers and citrus fruits, were developed in Japan. Then, continuous improvements were made to the performance of picking robots. A greenhouse cucumber picking robot was developed in the Netherlands, with a picking success rate of 74% and the picking time reduced to 50 s (Henten et al., 2003). An apple picking robot developed by Kyungpook National University, South Korea (Kondo and Ting, 1998) featured a manipulator with a workspace of up to 3 meters, a recognition rate of 85%, and a picking speed of 5 apples per second. In 2008, an automated picking system (Rath and Kawollek, 2009) for Gerbera jamesonii flower stalks was developed, and this objective was achieved through image analysis methods. During this period, research primarily focused on the feasibility verification of single function robots and the improvement of visual recognition and basic manipulation capabilities, laying an initial foundation for subsequent developments.

Since 2010, international picking robot has entered the commercialization phase. A strawberry picking robot designed for elevated substrate cultivation was developed by the National Institute of Agricultural Machinery (Hayashi et al., 2010). This robot had solved the common problems of low work efficiency, low success rate, fruit damage, difficulty in detection under complex lighting conditions, and high costs. An apple picking robot was developed by Sarabu et al. (2019). Dual robotic arms were employed in this robot for collaborative operation, which enhanced the recognition and picking success rates, and its performance in actual farm environments has gradually matured. In terms of intelligentization, the systematic integration of artificial intelligence, high resolution machine vision, and multiple modal sensor technologies has significantly enhanced the environmental perception and autonomous operation capabilities of robots in recent years. This stage marks the transition of picking robots from laboratory research to practical applications, with commercialization and intelligentization advancing in parallel, laying a solid foundation for the subsequent development of flower specific picking robots.

The above development trajectory indicates that picking robots have evolved from early single function devices into intelligent systems capable of adapting to complex agricultural environments. The continuous improvement in recognition accuracy, the ongoing optimization of actuators, and the progressive enhancement of system integration have jointly driven picking robots from laboratories toward practical production applications. This evolutionary trend provides important technological accumulation and valuable references for the development pathway of flower picking robots.

#### 2.1.2 Development of China’s flower picking robot

During the mid 20th century, the initial development of robot technology was initiated, and attempts were made by researchers to introduce this technology into the agricultural sector (Zhao et al., 2023). In China, the study of picking robots were began from the 1990s. Compared with developed countries of Japan and United States, China started relatively late in this field. At this stage, the research and development of the R&D of domestic flower picking robots mainly focused on learning and drawing. Gradually, the prototype development of some basic picking devices and simple control systems was carried out. However, there were still obvious limitations in terms of environmental adaptability, operation efficiency, and system stability. Overall, it is still in the stage of technical verification and initial exploration, and the actual application scope is relatively limited. During this period, Chinese flower picking robot research was primarily characterized by technology introduction and basic prototype development, with a focus on learning from foreign achievements, while significant gaps remained in system stability and environmental adaptability.

From the early 21st century to approximately 2010, targeted technological explorations for specific flower varieties began. The development of robot systems in controllable environments, such as greenhouses, initially achieved vision based target recognition, and basic mechanical picking functions were focused on in this stage (Yu et al., 2019). The complex growth environment and diverse shapes of flowers, as well as the requirement to avoid damaging flowers and plants during picking, however, the numerous technical bottlenecks were encountered in visual recognition and mechanical structure design, which restricted the performance and application of the robots (Bechar and Vigneault, 2016). With the rapid development of artificial intelligence, machine vision, and sensor technology, domestic flower picking robots have achieved key technologies. The edible rose picking robot was developed by Wang (2014). Rui et al. (2025) developed a honeysuckle harvesting robot that integrates high-precision visual recognition, adaptive positioning, and gentle picking mechanisms, achieving a field recognition accuracy exceeding 90% and a picking damage rate as low as 6%. This stage marks the transition of domestic flower picking robots from variety specific targeted exploration to breakthrough applications with integrated intelligent technologies, demonstrating enhanced capabilities in recognition precision, adaptive control, and damage reduction, and providing a replicable technical pathway for automated harvesting of other complex shaped flowers.

**Figure 3:**
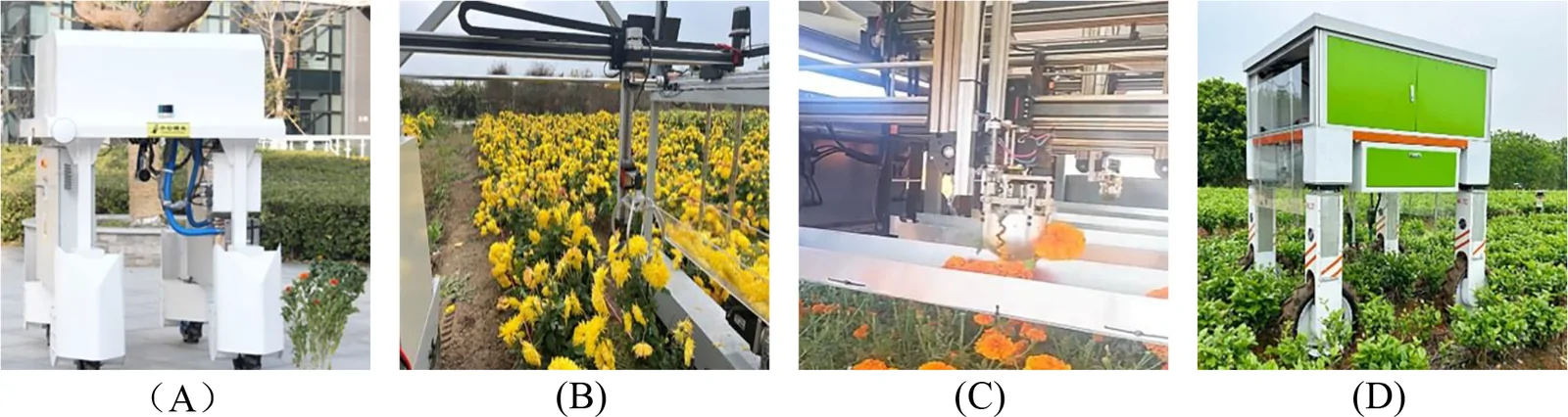
Representative flower picking robots developed for different species. **(A)** Safflower picking robot; **(B)** Golden chrysanthemum picking robot; **(C)** Marigold picking robot; **(D)** Jasmine picking robot.

The development trajectory of domestic flower picking robots has evolved from technology introduction and basic prototype development, through variety specific targeted exploration, to breakthrough applications with integrated intelligent technologies. The continuous advancement in visual recognition, mechanical design, and system integration has progressively narrowed the gap with international counterparts. Current systems have been developed for multiple flower species, including safflower, golden chrysanthemum, marigold, and jasmine, as shown in **Figure 3**. Yet significant challenges persist due to the considerable variation in flower size, morphology, and physical properties across these species. This evolutionary process provides valuable technological accumulation and practical experience for the future development of flower picking robots in China.

### 2.2 Analysis of development trend on picking robot

#### 2.2.1 Review methodology

To ensure the scientific rigor and reproducibility of this review, a systematic literature search and screening protocol was adopted. The Web of Science (WOS) Core Collection, Scopus, IEEE Xplore, and CNKI databases were queried using Boolean combinations of keywords, (“flower picking robot” OR “floral harvesting robot” OR “flower harvesting robot”) AND (“visual perception” OR “machine vision” OR “end effector” OR “robotic arm” OR “motion planning” OR “mobile platform”). The retrieval time span covered publications from 1985 to 2025, with the final search conducted in March 2025. Initial retrieval yielded 1,247 records. After removing duplicates and screening titles and abstracts, 312 articles were retained for full text review. The final corpus of approximately 120 references was selected based on the following criteria, relevance to robotic flower or high value crop picking, publication in peer reviewed journals or reputable international conferences, technical completeness with quantifiable performance metrics, and representation of key technological stages and geographic research contributions. The analytical framework guiding this review follows the perception, decision and execution paradigm, augmented by a Technology Readiness Level (TRL) assessment to contextualize the maturity of each reviewed technology.

#### 2.2.2 Analysis of development hotspots in picking robotic

**Figure 4:**
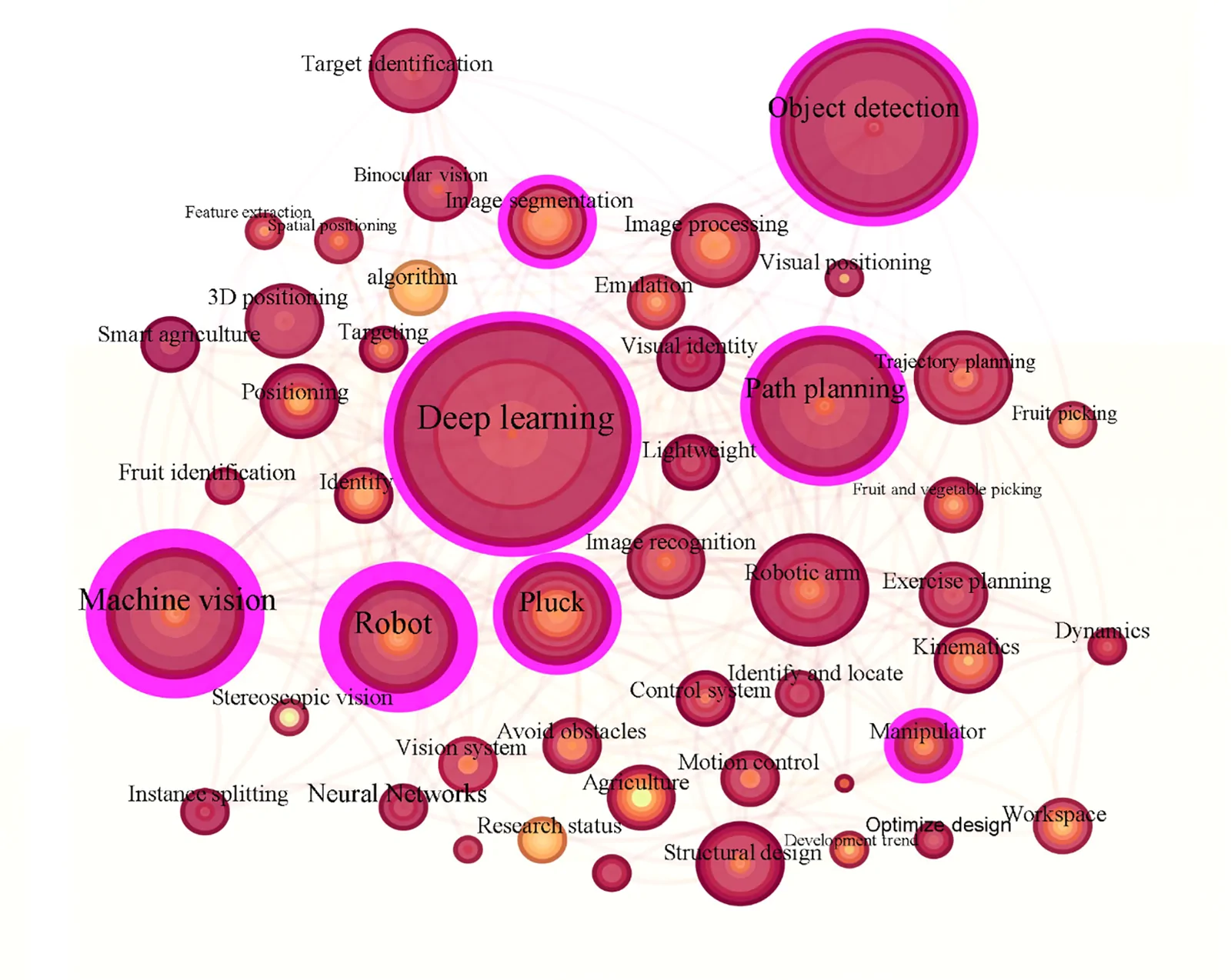
Hotspots of the picking robot.

Based on the literature corpus obtained through the systematic retrieval and screening protocol described in Section 2.2.1, a bibliometric analysis was conducted to identify the development hotspots and evolving trends in picking robotics research. Keyword co-occurrence analysis, cluster analysis, and temporal evolution mapping were performed using CiteSpace (version 6.3.R1), with parameters set as g-index (k=10), LRF = 2.5, LN = 10, LBY = 5, and e=1.0, generating 242 nodes and 815 links with a network density of 0.0279, a modularity Q of 0.3913, and a weighted mean silhouette S of 0.7337. indicating a well defined and reliable cluster structure. The current research on picking robots was closely revolved around the integrated technical route of perception to decision-making to execution. The target recognition and image segmentation in complex scenarios, autonomous path planning, and operation decision-making, the design and system control of compliant picking manipulators are focused on, as shown in **Figure 4**. These directions reflected the field of picking towards intelligence, precision, and systematization.

**Figure 5:**
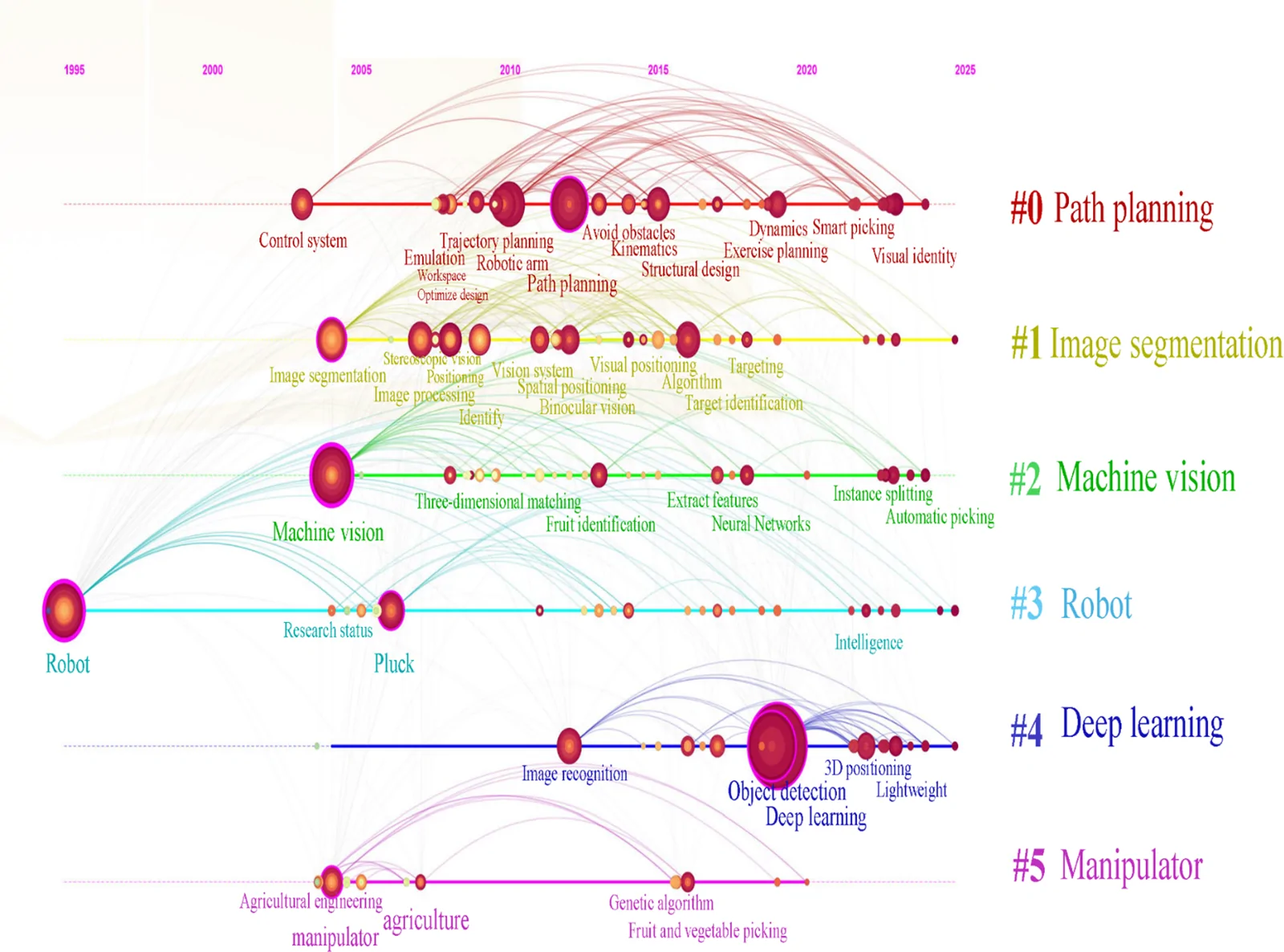
Hotspots in different time dimensions.

To study the dynamic changes in the research of picking robots, **Figure 5** reflects the research hotspots in different time dimensions. In the dynamic temporal framework, the evolutionary trajectory of research in the picking robot field could be explicitly traced. Around the year 2000, the technical framework for picking robots was established by foundational concepts, including control systems and machine vision. In 2010, the capabilities of “path planning” and “image segmentation” were gradually acquired by robots through specialized technologies such as control and vision systems. Since 2020, the perceptual and operational accuracy of picking robots has been substantially improved by the integration of intelligent technologies like deep learning and 3D positioning, as well as by optimizations, lightweight design, and genetic algorithms. Currently, this technology has been deeply anchored in agricultural scenarios, and its transition from basic research to practical field applications is symbolized by keywords such as agricultural robotic arms and fruit vegetable picking.

As a typical economic crop, the mechanized picking of flowers was indeed an important subfield of picking robot technology. The flower picking robot combined agricultural demands with intelligent equipment innovation, aiming to enhance the efficiency and quality of picking. Through visualization and multiple dimensional analysis of the literature in the WOS database, the core research direction in this field has been clarified. The directions of path planning, image segmentation, machine vision, robot, deep learning, and manipulator centrally reflected the technical key points and the main exploration paths. The solid basis and clear guidance for judging the development trends of the field and planning the subsequent research paths could be provided.

#### 2.2.3 Analysis of major contributing countries in picking robotic

Based on the literature corpus obtained from Section 2.2.1, a bibliometric analysis was conducted to examine the geographical distribution and collaboration patterns in picking robotics research. China, the United States, Japan, and the United Kingdom are the four most active contributors, collectively forming the core of the international collaboration network. China ranks first globally in publication output, accounting for approximately 60%. The United States ranks second, accounting for approximately 11%–18% of global publications. Japan ranks third, contributing approximately 6%–12%, with Kyoto University researcher Kondo N recognized as a pioneer since 1984. The United Kingdom ranks fourth, accounting for approximately 3%–5%, and serves as a key collaborator in international networks. Other European countries have also made significant contributions. As shown in **Table 1**.

**Table 1:** Major contributing countries and institutions in picking robotic.

Country	Publication volume (WOS)	Approximate share
China	1st globally	>60%
United States	2nd globally	11%–18%
Japan	3rd globally	6%–12%
United Kingdom	4th globally	3%–5%
Other countries		3%–5%

## 3 Key technologies of flower picking robot

### 3.1 Biological and environmental constraints and their impacts on robotic picking systems

The biological characteristics of flowers and the complexity of agricultural environments impose significant constraints on the perception, manipulation, and control capabilities of flower picking robots. Unlike industrial objects with fixed geometric structures and mechanical properties, flowers are living organisms with considerable variations in morphology, mechanical strength, growth stage, and spatial distribution. These biological variations directly affect the selection of picking strategies, the design of end effectors, and the operational accuracy of robotic systems. Compared with fruit, vegetable and crop harvesting, flower picking requires stricter control of contact force and positioning accuracy because visual appearance and structural integrity directly determine commercial value. Environmental conditions determine flower growth, development, and quality, thereby indirectly altering the physical properties that robots must interact with. Flower physical properties define the intrinsic mechanical interaction requirements between the robot and the target. These two aspects interact to determine robotic picking performance. Environmental conditions introduce external disturbances that affect perception reliability and operational stability, while simultaneously modifying flower physical properties that govern mechanical interaction. Understanding both aspects and their interrelationship is essential for improving the picking success rate and reducing flower damage.

#### 3.1.1 Environmental conditions

Environmental conditions encompass five primary factors that influence flower growth, development, and quality, namely light, temperature, humidity, gas composition, and fertilizer application. These factors collectively determine the morphological and mechanical characteristics of flowers that robots must perceive and interact with. Light intensity influences photosynthesis and growth, thereby determining flower size and structural development, while light quality influences flower color formation, directly affecting visual features used by recognition algorithms. Light period modulates flowering time, determining the growth stage of target flowers at harvest. Variations in illumination intensity and shadow patterns may reduce image quality and affect flower detection accuracy, and changes in light conditions alter flower color, shape, and texture features, thereby reducing model robustness under different operating environments. Temperature affects physiological metabolism, and extreme temperatures influence flower color formation and growth rate, modifying both the visual appearance and mechanical properties of floral tissues. Humidity affects transpiration and water absorption, influences disease occurrence, and alters tissue turgor and mechanical stiffness, which directly influence grasping force thresholds and cutting resistance during robotic picking. Gas composition, particularly CO_2_ and O_2_ levels, affects photosynthesis and respiration, thereby influencing the overall vitality and structural integrity of flowers. Fertilizer application provides essential nutrients, influences flower size and color quality, and affects plant health, with nutritional status directly correlating with tissue firmness and mechanical strength, thereby determining the operating force thresholds required for nondestructive picking.

#### 3.1.2 Physical properties

Flower physical properties are the direct consequence of environmental conditions and constitute the intrinsic constraints that determine robotic mechanical interaction. These properties can be divided into morphological characteristics and mechanical characteristics, which are intrinsically coupled. Morphological characteristics include flower size, stem orientation, peduncle position, and spatial distribution. These parameters exhibit substantial variability across different species, growth stages, and even individual plants within the same cultivar, primarily because environmental conditions create heterogeneous growth environments. Bloch et al. (2018) and Zhongliang et al. (2021) emphasized that such structural variations significantly influence robotic picking performance, increasing uncertainty during target detection and grasping. In practical applications, the difference between the detected flower position and the actual picking point can result in positioning errors, leading to grasping failure, collision, or flower damage. Therefore, visual localization accuracy and motion compensation capability should be designed according to the biological variability of target flowers.

Mechanical characteristics encompass the structural support, load bearing capacity, and resistance to bending and breaking of plant organs. The biological characteristics of plants directly determine the adaptability of picking methods, the threshold of operating force, and the degree of crop damage (Zhang et al., 2020a). Different picking methods, including grasping, twisting, and cutting, require specific mechanical parameters of flower stems and peduncles. Liu et al. (2008) investigated the mechanical properties of tomatoes for robotic harvesting and reported an average peduncle breaking moment of 161.29 N·mm and an average tensile force of 22.53 N, concluding that bending is more suitable than stretching for robotic picking. Rabbani et al. (2015) analyzed the mechanical properties of Damask roses and reported a mass density module ranging from 0.53 to 6.3 MJ, a crush resistance coefficient ranging from 0.38 to 9.5 N/mm, and a cutting efficiency coefficient ranging from 0.86 to 0.99. The study demonstrated that increasing the cutting bevel angle from 0° to 45° improved cutting efficiency from approximately 0.95 to 0.98, indicating that cutting geometry significantly affects harvesting performance. These results highlight that cutting mechanism parameters, material selection, and actuator output should be optimized according to the mechanical characteristics of flower stems. Critically, these plant specific mechanical parameters should be translated into engineering design inputs. For example, cutting force and stem mechanical strength can provide references for determining actuator output, blade geometry, and cutting strategies. Similarly, compression properties and peduncle stiffness can guide the selection of grasping pressure and compliance characteristics of soft robotic fingers. Such quantitative relationships between plant biomechanics and robotic design parameters are essential for achieving efficient and nondestructive flower picking. Without such quantitative mapping from plant biomechanics to engineering specifications, end effector designs are prone to either excessive conservatism that compromises operational efficiency, or inadequate compliance that results in mechanical damage to floral tissues.

Environmental conditions and flower physical properties are not independent. Environmental conditions are the root cause of variations in flower physical properties. Light, temperature, humidity, gas composition, and fertilizer collectively determine the morphological and mechanical characteristics that robots must perceive and interact with. Environmental conditions can directly or indirectly alter flower physical properties, thereby changing the mechanical interaction requirements. For instance, light intensity and quality influence flower color formation and development (Daqiu and Jun, 2015), which modifies visual features used for recognition. Temperature and humidity affect tissue turgor and mechanical stiffness, thereby changing grasping force thresholds. Shading conditions influence peony growth and flower quality (Zhao et al., 2012), modifying both morphological parameters such as size and shape and mechanical properties such as tissue firmness. Fertilizer application determines nutritional status, which directly correlates with flower size, color quality, and tissue mechanical strength. Flower physical properties determine how environmental disturbances propagate through the robotic system. Flowers with slender, flexible stems exhibit larger amplitude oscillations under wind disturbance compared to those with rigid, thick stems, thereby amplifying the localization challenge. Similarly, dense flower clusters, a morphological characteristic, exacerbate occlusion problems under variable illumination, whereas sparse distributions may suffer more from background clutter and false detection. Environmental factors, including illumination conditions, wind induced plant motion, flower spatial distribution, and leaf shading, significantly influence robotic picking performance (Kun et al., 2022). These factors should not only be considered from the perspective of plant growth but also as external disturbances affecting robot perception, localization, and execution. Variations in illumination intensity and shadow patterns may reduce image quality and affect flower detection and segmentation accuracy. Wind induced movement of stems and flowers causes dynamic changes in target position, increasing localization errors and requiring real-time visual feedback and motion compensation. Moreover, dense foliage and overlapping flowers create occlusion problems, reducing available visual information and increasing the difficulty of path planning and collision avoidance.

**Figure 6:**
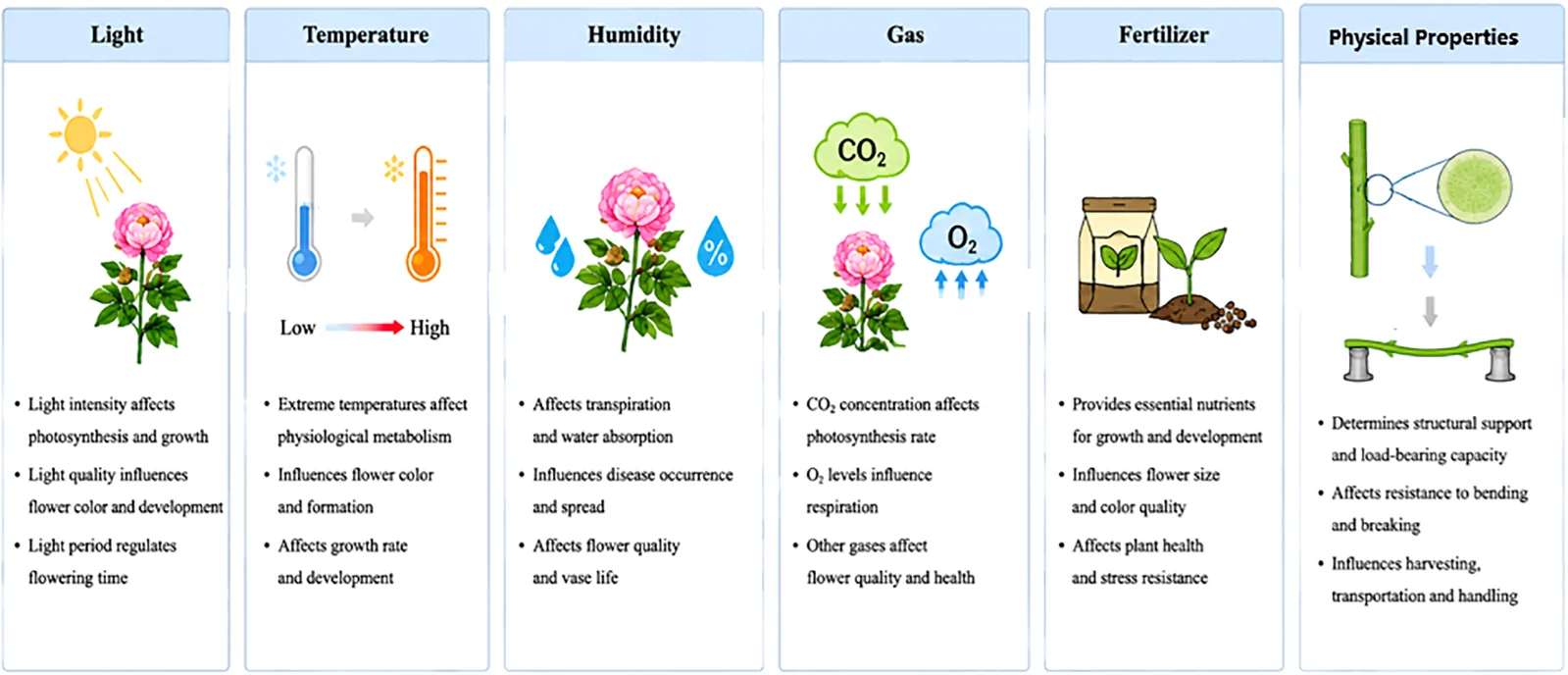
Environmental factors and physical properties affecting flower picking robots.

Therefore, environmental conditions and flower physical properties should be integrated into the design framework of flower picking robots. Environmental conditions are the root determinants of flower physical properties, while simultaneously introducing external disturbances that affect perception reliability, positioning accuracy, and operational stability. Biological parameters determine the mechanical interaction requirements between the robot and flowers. The influence mechanism from environmental conditions and biological characteristics to robot perception, localization, end effector operation, and picking performance is illustrated in **Figure 6**.

### 3.2 Vision system for target perception and localization

In recent years, flower picking robots have made significant progress in target perception and recognition, three-dimensional space construction and positioning, and decision-making planning, driven by advances in artificial intelligence technology (Redmon et al., 2016). Various perception approaches differ significantly in recognition capability, localization accuracy, computational efficiency, and deployment requirements. Therefore, this section presents a comparative analysis of representative perception technologies, categorized into object detection, instance segmentation, three-dimensional localization, and multimodal perception, as summarized in **Table 2**.

**Table 2:** Comparison of perception technologies for flower picking robots.

Perception method	Main output	Performance characteristics	Application in robotic picking
Object detection	Target location	Fast inference, low model complexity, moderate localization accuracy	Suitable for rapid target searching, but limited for precise picking under occlusion
Instance segmentation	Pixel-level target information	High recognition accuracy and localization precision, but higher computational cost	Suitable for flower contour extraction and accurate picking point estimation
3D localization	Spatial coordinates	High positioning accuracy, but increased system complexity	Provides essential information for robotic arm positioning and manipulation
Multimodal perception	Fused multiple source information	Improved robustness with higher computational requirements	Enhances adaptability under complex environments

Object detection methods provide advantages in processing speed and system deployment, making them suitable for rapid target searching in complex agricultural environments, with technical support furnished for harvesting decision-making and path planning (Xiuling et al., 2024). Their main advantage is high inference speed and relatively low computational requirements, making them suitable for real-time robotic applications. Liu et al. (2020) combined the Otsu algorithm to convert the RGB color space to the HIS color space, obtained thresholds, and effectively identified targets to be picked. Zhao et al. (2021) improved YOLOv3 as the backbone network and designed an adaptive feature reconstruction recognition algorithm, which achieved a mean Average Precision of 96.3% and an F1 score of 91.8%, outperforming the original YOLOv3 by 3.8% in both metrics, with detection speed reaching 27.8 FPS on GPU. These methods generally provide only bounding box information, which limits the accurate estimation of flower contours and picking points, especially under conditions involving overlapping flowers and severe occlusion (Yang et al., 2023). Therefore, detection accuracy alone cannot fully represent the suitability of a perception method for robotic picking tasks, and localization error and real-time performance should also be considered.

Instance segmentation methods improve target boundary extraction and localization accuracy by providing pixel level information, which is more suitable for estimating flower boundaries and determining grasping regions. This advantage is particularly important for flowers with irregular shapes and dense distributions. Semantic segmentation was applied to picking robots for accurate identification and localization of target picking objects (Wen et al., 2020). It could be used in target localization, maturity recognition, target differentiation, obstacle identification, and other aspects, improving the perception ability and picking accuracy of picking robots. Xue et al. (2019) achieved classification and identification of 20 Chinese medicinal plant species using two ANN-based methods, with overall accuracies of 98.3% and 92.5%, respectively. Segmentation models usually contain more parameters and require higher computational resources, which may reduce inference speed and limit deployment on embedded robotic platforms.

Three-dimensional localization technologies provide essential spatial information for robotic manipulation by acquiring 3D coordinates of targets, thereby providing positional information for robotic arm grasping and directly supporting robotic arm positioning and end effector adjustment. Technologies such as depth estimation and localization, global map construction, and scene segmentation constitute the core components of the visual system (Rasheed et al., 2024). The construction of a global map of the operating scene enables autonomous navigation. Scene segmentation is utilized to distinguish between fruits, branches, leaves, and obstacles, thereby supporting obstacle avoidance and path planning. The performance of depth-based methods can be affected by sensor cost, calibration errors, illumination variation, and outdoor environmental conditions.

Multimodal perception integrates multiple sensing modalities to enhance the robustness of visual systems. In addition to traditional visual algorithms, near infrared and spectral technologies can be applied to the recognition and positioning of picking robots (Ye et al., 2020). In pixel-based classification, the overall accuracies of SVM, RF, and ANN reached 91.4%, 90.0%, and 91.1%, respectively, with Kappa coefficients of 0.80, 0.77, and 0.79. The inclusion of the red edge band improved the overall accuracy by 2.9% to 3.0%. The future trend is moving towards the integrated development of multimodal perception to enhance the robustness of visual systems. Furthermore, recent advances in deep learning architectures are opening new avenues for agricultural vision systems. Vision Transformer-based models have demonstrated superior capability in capturing global feature dependencies compared to conventional CNNs, making them more robust to occlusion, background clutter, and variable lighting conditions. The self-attention mechanism enables the model to focus on discriminative floral features while effectively suppressing distracting background information. The emergence of large scale foundation models pretrained on vast image corpora has enabled few shot and zero shot recognition capabilities, which is particularly valuable for flower picking applications where hundreds of commercial varieties exist and collecting large labeled datasets for each is economically prohibitive.

Overall, the selection of perception technologies for flower picking robots should consider the trade-off among accuracy, computational efficiency, environmental adaptability, and system integration. Object detection is more suitable for rapid target searching, segmentation methods are beneficial for precise localization, and 3D perception provides essential spatial information for manipulation. Future systems should combine multiple sensing modalities with lightweight algorithms to achieve robust perception under dynamic agricultural environments.

### 3.3 Robotic arm configurations and its motion planning

A robotic arm is an automated device that can achieve multipledegree of freedom movement in space through instruction programming as the core executive component of robot technology (Tai et al., 2016). Meanwhile, it can also complete precise positioning and operation tasks. In the field of agricultural picking robots, the structural design of the robotic arm directly determines the positioning accuracy, movement speed, and load capacity of its end effector (Blanes et al., 2011). It profoundly affects the adaptability of the overall system to complex crop shapes, randomly distributed targets, and flexible picking operations, thus becoming a key technical challenge for improving picking efficiency and ensuring operation reliability. Therefore, the study of the structural optimization design of a robotic arm for agricultural picking is of great significance for promoting the automation and intelligence of agricultural production (Erfani et al., 2019).

#### 3.3.1 Robotic arm configurations

According to the number of robotic arms working collaboratively in the system, agricultural picking robots can be classified into single arm, dual arm, and multiple arm systems. Their design choices directly correspond to different levels of operational complexity, work efficiency, and cost.

**Figure 7:**
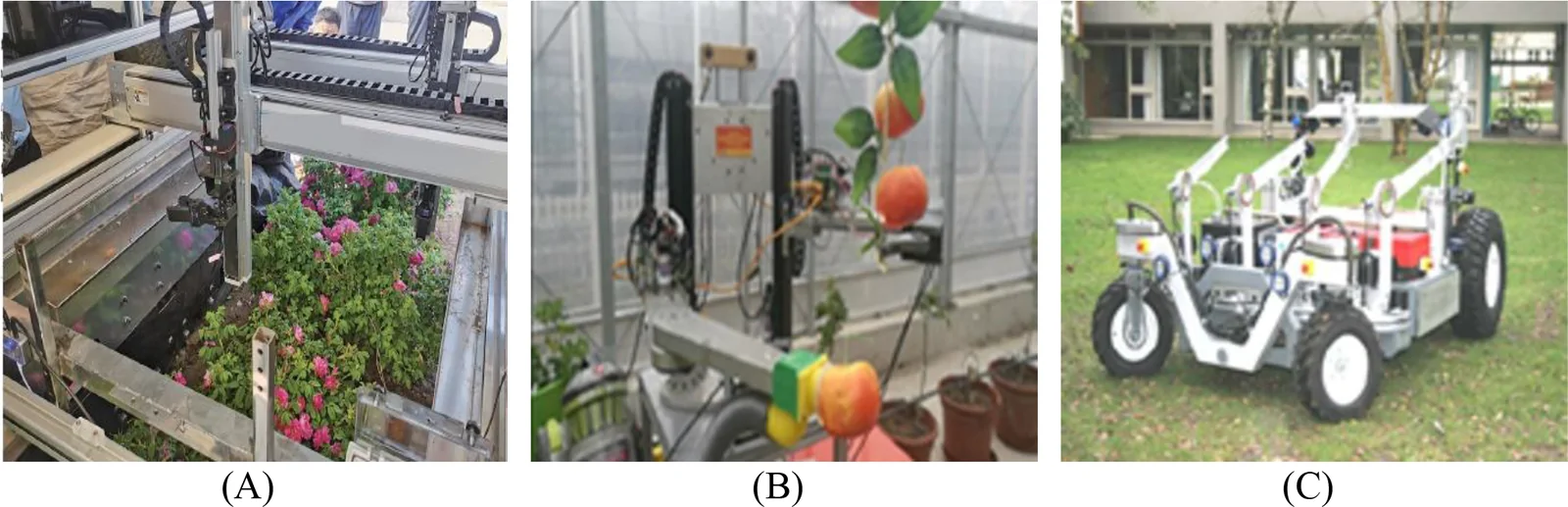
Robotic arm configurations. **(A)** Single arm robot arm for picking rose; **(B)** dual arm robot arm; **(C)** Multiple arm robot arm.

Single arm systems adopt a 4–6 degree of freedom serial configuration, mainly driven by electricity, with a customized end effector integrated at the end. Its structure is relatively simple, the control logic is clear, and the cost is low. It is suitable for scenarios with sparse targets and strong sequentiality of operations. Their limited workspace and single manipulation capability may restrict efficiency when dealing with dense target distributions or complex plant architectures. Tang et al. (2021) studied the six link manipulator used in a citrus picking robot. Its motion planning time was reduced by 54.89% compared with the traditional APF, and the total joint error was decreased by 45.41%. The planning time for a single picking path was only 0.40 s, and the total planning time for the entire process was 2.56 s, fully validating its real-time advantages under the synergy of visual modeling and motion planning. Recent developments in rose intelligent picking robots have demonstrated the potential of single arm systems for flower harvesting applications (**Figure 7A**). For flower picking applications, robotic arms require additional capabilities because flowers generally possess fragile petals and slender peduncles. Accurate positioning, compliant interaction, and low-force manipulation are essential to reduce mechanical damage during picking.

Dual arm systems significantly enhance operational flexibility and efficiency through the coordinated control or task allocation of the two robotic arms. Xiao et al. (2018) developed a dual arm picking robot for tomatoes, which ensured the stability of the operation through the coordinated cooperation of the two arms. The picking success rate of this robot can reach 87.5%, and the average picking time for a single tomato is approximately 29 s (**Figure 7B**). Takeshi et al. (2022) developed a dual arm picking system for V-shaped trellis cultivation environments. Compared with single arm systems, dual arm configurations provide expanded workspace and enhanced adaptability to complex plant structures. The introduction of a second arm significantly complicates motion planning due to the need for real-time inter arm collision avoidance and coordinated trajectory synchronization. The two arms share overlapping workspace in dense canopies, where mutual interference and collision risks increase substantially.

Multiple arm systems are complex operation units composed of three or more robotic arms, used for large scale and time pressured harvesting tasks with high spatial coverage and parallel operation capabilities. Scarfe et al. (2009) designed a kiwifruit picking robot with four 3 degree of freedom arms, achieving a picking speed of 1 fruit per second (**Figure 7C**). Multiple arm synchronous operations significantly improve picking efficiency compared with single arm and dual arm systems. Motion planning complexity increases exponentially with arm number. Each additional arm introduces new degrees of freedom and interference constraints, requiring global task allocation and local collision avoidance in overlapping workspaces. Their application in flower picking remains limited due to system complexity, hardware costs, and coordination demands.

#### 3.3.2 Motion planning for multiple arm systems

Motion planning is a key factor influencing the efficiency and reliability of robotic picking operations. The choice and complexity of motion planning methods are closely related to the number of robotic arms in the system. Existing motion planning methods can be divided into traditional approaches and intelligent optimization methods.

Traditional algorithms, including artificial potential field and rapidly exploring random tree, provide efficient solutions for collision avoidance and path generation due to their simple implementation and low computational cost. For single arm systems, APF is computationally efficient and suitable for real-time obstacle avoidance, but suffers from the local minimum problem in dense flower canopies. RRT excels at finding feasible paths in high dimensional configuration spaces, yet its stochastic nature produces suboptimal path lengths and lacks asymptotic optimality. For dual arm systems, the configuration space doubles and traditional algorithms struggle with the curse of dimensionality, while self-collision avoidance between arms further complicates path generation. For multiple arm systems, computational complexity becomes prohibitive as the joint configuration space grows exponentially with arm number. Improved variants such as RRT* address these limitations through path optimization and targeted sampling, yet their performance may decrease in dynamic agricultural environments with changing obstacles and uncertain target locations.

Intelligent optimization methods, such as reinforcement learning and evolutionary algorithms, improve adaptability by learning complex environmental information. These methods show particular promise for multi arm coordination. Reinforcement learning-based planners offer greater adaptability to dynamic disturbances such as wind induced target motion. For dual arm systems, reinforcement learning can learn cooperative strategies that account for inter arm constraints. For multiple arm systems, distributed learning enables each arm to develop collision-aware policies while contributing to global task objectives. The high training cost and sim-to-real transfer challenge remain significant barriers. Hybrid approaches combining the global planning capability of RRT with the local adaptation of learning-based methods may offer the most promising pathway for flower picking applications. Experimental studies have demonstrated the effectiveness of improved planning algorithms. Yan et al. (2024) proposed a 3D path planning algorithm for obstacle avoidance harvesting that optimizes initial pheromone distribution. The success rates reached 96%, 86%, and 92%, with planning time reduced by 49.38%, 46.33%, and 51.03% compared with the traditional ant colony algorithm. Guo et al. (2024) developed an improved RRT algorithm based on target-biased parent node selection for obstacle avoidance in complex environments. In both static and dynamic environments, the average planning time of the S-RRT algorithm was 242.2 ms and 79.4 ms, respectively, a significant improvement over traditional RRT. It was capable of rapidly replanning paths after dynamic obstacles appeared, completing joint angle adjustments within approximately 2.5 seconds (Wei and Ren, 2018).

Training data, computational resources, and real-time deployment requirements remain important challenges. Future flower picking robots should integrate perception information, obstacle avoidance strategies, and adaptive motion planning methods to achieve efficient and reliable autonomous operation in unstructured environments.

### 3.4 End effector for non-destructive grasping

**Figure 8:**
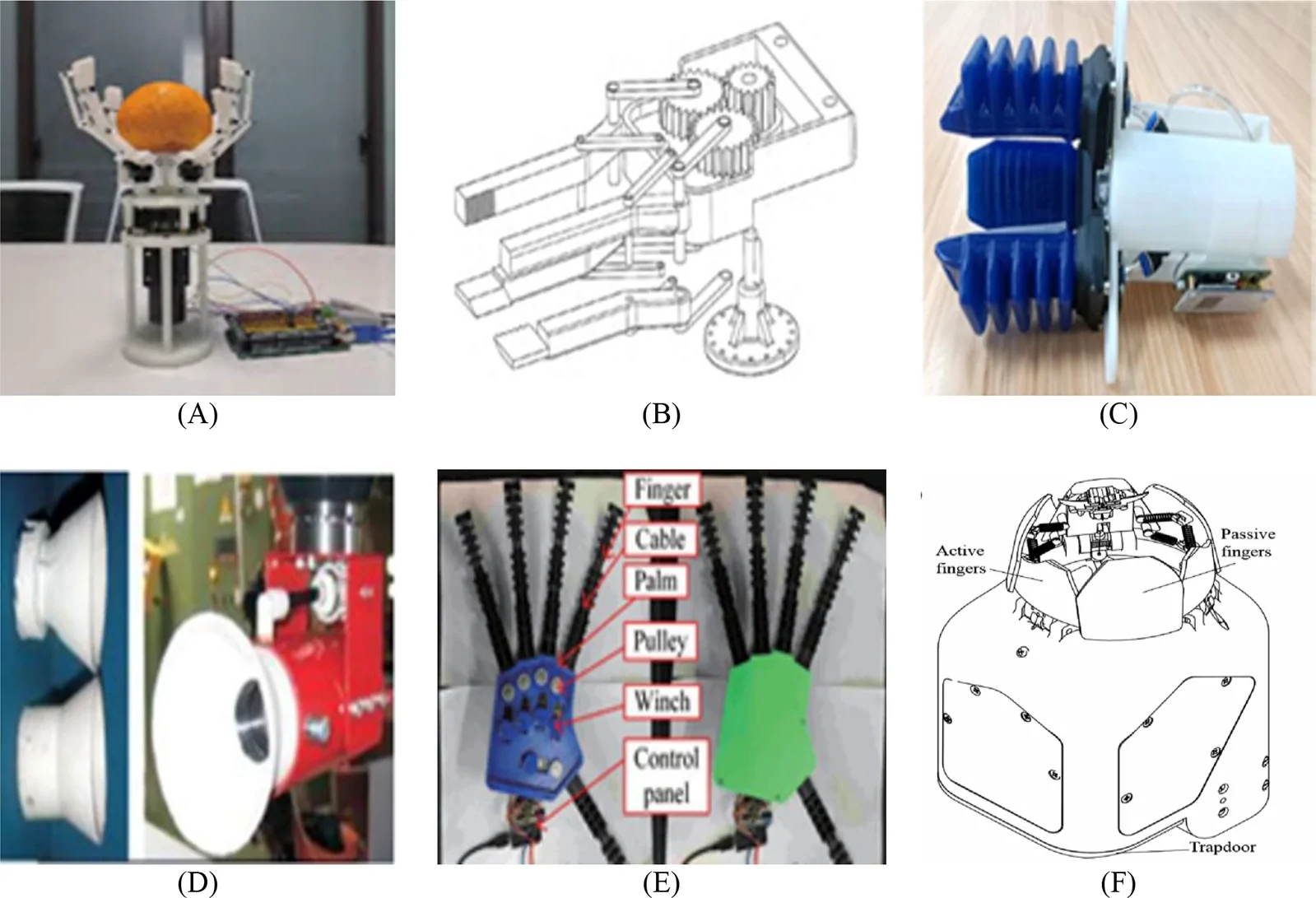
Representative end effectors used in agricultural picking robots. **(A)** Underactuated joint-type end effector; **(B)** Integrated clamping and cutting device; **(C)** Flexible grasping pneumatic three-finger structure; **(D)** Suction-cup end effector; **(E)** Humanoid rope driven soft gripper; **(F)** Rope driven gripper.

The flower picking robot is recognized as core equipment for realizing automated and intelligent picking of high value flowers. As the robotic “hand” responsible for direct interaction with floral targets, the end effector directly determines picking success rate, operational efficiency, and post-harvest flower quality (Wu et al., 2021a).The end effectors are mainly classified into mechanically driven, pneumatically driven, and rope driven types. Representative examples of these three categories are presented in **Figure 8**, while their characteristics and applicability to flower picking scenarios are summarized in **Table 3**. Compared with fruit, vegetable, crop harvesting, flower picking requires more compliant interaction due to the fragile petals, slender peduncles, and irregular structures of floral targets. Mechanical damage during picking is mainly related to excessive contact force, inappropriate grasping position, insufficient contact area, and rigid interaction between the end effector and the target. End effector design should balance grasping stability and mechanical compliance to minimize damage while maintaining operational efficiency.

**Table 3:** Characteristics of the three types of end effector.

Types of end effectors	Driving mechanism	Advantages	Limitations	Application in flower picking
Mechanically driven end effector	Motor as a power source, driven by a transmission structure	High positioning accuracy; precise cutting; fast response	Rigid contact may cause damage to fragile petals and stems	Flowers requiring precise stem cutting
Pneumatically driven end effector	Air pressure controlled deformation and grasping	Good flexibility, stable output force	Additional air supply system; complex control	Soft flowers and irregular structures
Rope driven end effector	Rope tension controlled flexible deformation	Lightweight; high flexibility; low damage potential	Nonlinear control and rope wear	Fragile flowers requiring gentle grasping

At present, there are still some technical issues to be addressed. Different types of end effectors are required for low damage and efficient flower picking in response to varied operating environments and scenarios (Zhang et al., 2010).

Mechanically driven end effectors exhibit high positioning accuracy and fast response for heavy target objects requiring high precision harvesting. Yu et al. (2023) designed an underactuated joint-type end effector for citrus picking (**Figure 8A**). The end knuckle is equipped with a sponge, which can protect the fruit and increase friction. Orchard tests yielded a 92.9% success rate for 68–106 mm citrus, with an average picking time of 7.3 s per fruit (3.2 s for enveloping, 4.1 s for twisting) and a damage rate of 5.9%. This end effector is suitable for picking targets that are prone to damage. Wang et al. (2021) developed an integrated clamping and cutting device for rose picking scenarios (**Figure 8B**). Through gear meshing, the two clamping jaws rotate to hold the rose stem. At the same time, the tool holder under the clamping jaws drives the flat blade to mesh with the serrated blade, completing the cutting of the rose stem while the jaws hold it. Mechanically driven end effector exhibits picking advantages for certain heavy target objects requiring high precision harvesting. For flower picking applications, the high stiffness and rigid contact characteristics of mechanically driven structures may increase the risk of petal deformation and stem damage. Optimization of gripping force, contact surface design, and cutting position is important for achieving precise and nondestructive flower picking. Pneumatically driven end effectors offer adaptive deformation and adjustable contact force, providing valuable references for flower picking robots because both applications require gentle interaction and damage reduction. Chen et al. (2022) designed a pneumatic three-finger structure, which also integrates a small air supply device to achieve flexible grasping (**Figure 8C**). The pneumatic flexible end effector can reach an air pressure of 30 kPa within 3 seconds during clamping, with the opening action completed within 2 seconds. The overall prototype weighs approximately 1 kg. To maintain the integrity of the picked targets’ appearance, Baeten et al. (2008) developed a suction-cup end effector specifically for apple picking (**Figure 8D**). Its core working principle lies in using the negative pressure suction generated by a vacuum pump to stably adsorb the apple. Abundant Robotics (U.S.) (Li et al., 2019) has developed and designed an apple picking robot. This robot uses vacuum technology to suck targets into a collection box, featuring high picking efficiency (Zhang et al., 2020b). Zhao et al. (2019) designed and verified a multiple degree of freedom fruit and vegetable picking manipulator based on a pneumatic flexible actuator. A pneumatically driven end effector is typically used for handling relatively soft objects. Their adaptive deformation and adjustable contact force provide valuable references for flower picking robots because both applications require gentle interaction and damage reduction. Flowers have lower tolerance to mechanical stress due to their fragile petals and delicate structures.

Rope driven end effectors feature high compliance and lightweight characteristics, making them suitable for flower picking scenarios requiring adaptive wrapping and low force interaction. Sung and Joe (2025) proposed a robotic manipulator that can use a wire driven variable link mechanism to control its workspace. Wu et al. (2021b) designed a new type of rope driven soft gripper, which is similar to a human hand in both shape and function (**Figure 8E**).FEM results show that the finger with the largest material removal achieved a maximum deformation of 93.623 mm, an increase of approximately 155% compared to the original design (36.668 mm). Xiong et al. (2019) adopted a rope driven structure (**Figure 8F**). Resulting in gentler transmission that avoids crushing damage to strawberries caused by rigid drive. Rope driven end effector features greater flexibility in working environments while minimizing damage to objects. The high compliance and lightweight characteristics of rope driven mechanisms make them suitable for flower picking scenarios requiring adaptive wrapping and low force interaction. By adjusting rope tension and structural deformation, these end effectors can reduce localized pressure on petals and improve the safety of delicate target handling.

### 3.5 Mobile platform with diverse cultivation environments

Flower picking is identified as a critical procedure in agricultural production, featuring strong timeliness, high labor intensity, and complex operational settings including greenhouses and open fields. Automated picking robots are recognized as core technologies for addressing labor shortages and enhancing harvesting efficiency and product quality. The mobile platform, acting as the legs of such robots, is tasked with the transportation of picking actuators, perception systems, control systems, and energy supply systems. Autonomous navigation, positioning, and stable operation in complex and variable flower cultivation environments are enabled by the mobile platform, with its performance directly governing the overall operational efficiency and environmental adaptability of the robot. Compared with general agricultural robots, flower picking robots impose higher requirements on mobile platforms due to the fragility of floral targets, dense planting patterns, and strict positioning requirements. Mobile platforms should not only provide sufficient mobility and load capacity but also maintain stable motion to support accurate visual perception and robotic manipulation. To maintain operational stability, mobile platforms are required to exhibit specific obstacle surmounting capabilities. Centimeter level precision in autonomous navigation and positioning must be achieved in densely planted areas, ensuring the robotic arm can be accurately maneuvered to the vicinity of target flowers. In addition, sufficient load bearing capacity must be provided to support the robotic arm, vision system, controller, power supply, and potential harvesting containers, while flexible steering and movement in narrow spaces are enabled simultaneously. Mobile platforms are primarily categorized into three types, namely wheeled platforms, tracked platforms, and rail mounted platforms.

**Figure 9:**
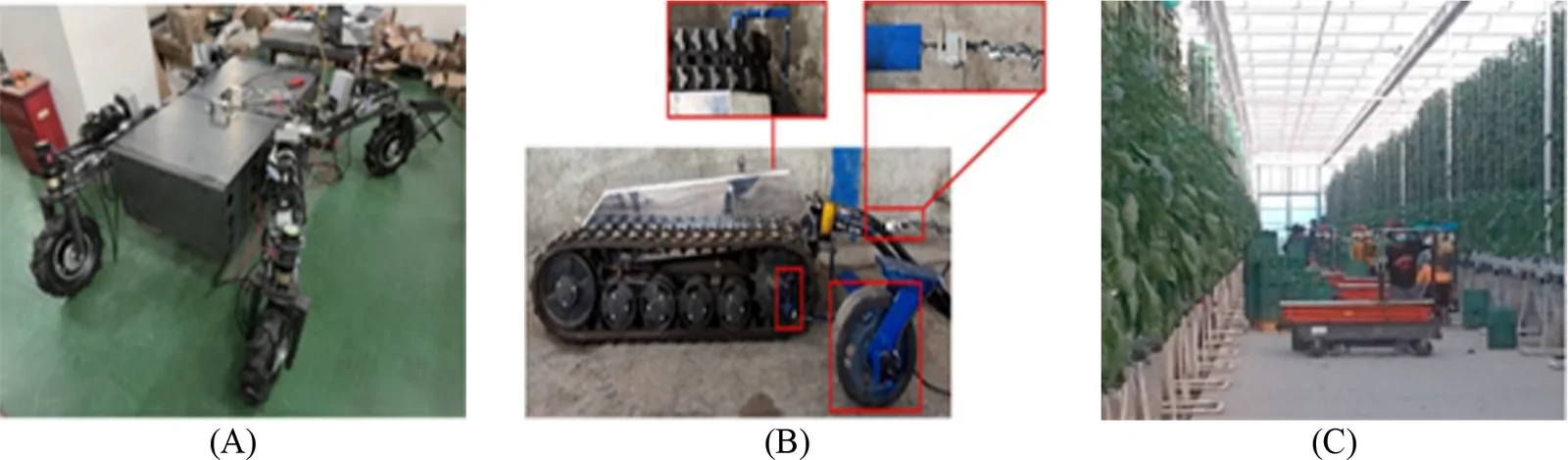
Different types of mobile platforms. **(A)** Bionic wheeled chassis; **(B)** Full track chassis; **(C)** Rail mounted platforms.

Wheeled platforms feature a relatively simple structure, mature reliability, low cost, fast movement speed, relatively low energy consumption, and good maneuverability. Due to their high mobility and flexible steering capability, wheeled platforms are widely used in greenhouse flower picking environments with relatively flat terrain and regular planting layouts. Their obstacle crossing capability and stability may be limited in uneven outdoor environments. In greenhouse environments, wheeled platforms have become mainstream due to their flexibility and efficiency. Pan et al. (2024) designed a chassis integrated with bionic mechanical for hilly and mountainous areas (**Figure 9A**). The NSGA-II-based leveling control achieved a maximum pitch error of 1.08° and roll error of 1.19°, with standard deviations of 0.216° and 0.176°, outperforming PID control. Harik and Korsaeth (2018) designed a wheeled mobile logistics robot. In greenhouse environments with limited signals, the robot can estimate its position and achieve autonomous navigation and active obstacle avoidance.

The track plates of tracked platforms rotate cyclically, which greatly reduces ground pressure. They have strong terrain adaptability and are suitable for heavy loads and traction. Compared with wheeled platforms, tracked platforms generally provide stronger stability, higher load bearing capability, and better obstacle crossing performance, making them suitable for complex outdoor flower fields and uneven agricultural environments. Their movement speed and energy efficiency are relatively limited. Gao et al. (2020) designed a full track modular unmanned agricultural power chassis and a full track vehicle travel system. This system enables in place steering of the driving chassis and exhibits good stability (**Figure 9B**). The chassis features a 410 mm ground clearance, 45° approach angle, 30° departure angle, and 35°climbing capability.

Rail mounted platforms can travel safely, stably, and at high speeds on dedicated rails. Due to their constrained trajectories, rail mounted platforms exhibit high positioning stability and low motion disturbance, which is beneficial for vision-based localization and precise robotic arm operation. Their application is restricted by the requirement for fixed infrastructure. Their operation paths are strictly limited, making it easy to achieve automation and centralized scheduling. EuTteum and DaeYeong (2022) designed a two-wheel-driven mobile robot that operates with smart farm rails and corridors (**Figure 9C**). The chassis supports 350 kg, with no deformation under 450 kg centralized load, and a safe front load limit of 370 kg.

Overall, different mobile platforms exhibit different advantages in terms of movement speed, load capacity, terrain adaptability, and motion stability. Wheeled platforms are more suitable for structured greenhouse environments requiring high mobility, tracked platforms provide better adaptability for complex terrains, and rail mounted platforms are advantageous in scenarios requiring high positioning accuracy and operational consistency. Future flower picking robots should focus on the coordinated optimization of mobility, stability, and compatibility with perception and manipulation systems.

## 4 Application environments of flower picking robots

The design paradigm, technical performance, and ultimate applicability of flower picking robots are deeply coupled with their specific working environment. Based on the main spatial forms of modern flower production, the application scenarios can be macroscopically divided into open field environments and facility controllable environments. These two types of environments have essential differences in spatial structure, climatic conditions, planting systems, and production processes, which put forward completely different technical requirements for the robot’s subsystems of perception, decision-making, execution, and movement, as shown in **Table 4**.

**Table 4:** Comparison of technical requirements for robotic subsystems between the two environmental types.

Type	Research features	Research subject and characteristics	Focus of research	References
Open field environments	Environmental perception and target recognition	Convolution-free ViT Backbone Network	Accurate and instant chrysanthemum counting, an unstructured application	(Qi et al., 2025)
		Complex unstructured environments and high precision detection	Precise fruit detection, picking point localization	(Yu et al., 2019)
		Continuous closed loop visual servo control system	Integrated control of positioning, picking	(Chen et al., 2024b)
		Proposing an enhanced RRT algorithm	The improved artificial potential field method, to optimize the guidance of sampling points	(Fang et al., 2025)
	Execution and grasping	Three novel silicone-based end effectors	Novel end effectors, to enhance the picking performance	(Lu et al., 2022)
		Modular pneumatic end effector	Rapid non-destructive harvesting	(Ochoa and Mo, 2025)
		Control method for parallel dual arms	Integration of movement, observation, and harvesting	(Zhang et al., 2025b)
		Two picking system architectural solutions	Solve the singularity problem in complex working environments	(Lei et al., 2025)
	Path planning and navigation	Novel hybrid powered mobile platform	Using the platform to improve picking efficiency and working conditions	(Ferreira et al., 2018)
		A real-time receding horizon estimation and control framework	High precision path tracking under uneven and moist soil conditions	(Kayacan et al., 2018)
		Local dynamic path planning, advanced motion control	Address energy constraints, dynamic individual interaction, and complex terrain	(Eiffert et al., 2020)
		Evaluate the effect of optimizing the field operation path planning	Combining machine vision and GPS technologies, automatic navigation of vehicles	(Rodias et al., 2017)
	Biological characteristics	Sowing date and irrigation regime for safflower	Physiological properties, yield, and water use efficiency	(Bahadori et al., 2025)
		Moderately shaded intercropping system for chrysanthemum	Determine the optimal intercropping pattern	(Yang et al., 2024)
		Evaluate the impact of heterogeneity on in field plant diversity	Enhance in field plant diversity by increasing the heterogeneity of crop mosaics	(Alignier et al., 2020)
		Mechanisms for improving photosynthetic yield of complex light environments	Daily high light intensity continuously enhances photosynthesis	(Wu et al., 2023)
Facility controllable environments	Environmental perception and target recognition	Three-level ripeness classification and 6D pose estimation of fruits	Picking motion control algorithm, efficient, precise, and non-destructive picking operations	(Kim et al., 2022)
		A lightweight S-YOLO model	High precision and high speed detection of tomato fruits	(Sun, 2024)
		A kind of inverse kinematics method based on the arm angle	Optimize the joint analysis of the picking robot	(Rong et al., 2024)
		Dual view detection and real-time optimization of grasping posture	Low damage and high efficiency picking decision-making	(Dai et al., 2024)
	Execution and grasping	Passive cut flower end effector tested in greenhouses	Dedicated end effector, verify harvest success rate	(Sytsma et al., 2025)
		Optimize the link lengths and joint angles of the robotic arm	Overcomes the problems of low flexibility and singularity proneness in conventional robots.	(Al-Khulaidi et al., 2022)
		A multiple joint robotic arm with cutting and grasping mechanisms	Identify the optimal picking points and estimate the contour area of the fruits	(Chang and Huang, 2024)
		“One-eye and two-arms” high-speed visual servo system	Develop a fuzzy fruit stalk localization algorithm	(Jiang et al., 2022)
	Path planning and navigation	Compact, low cost, and high precision wheeled chassis	Chassis motion control and positioning accuracy	(Su et al., 2023)
		Flexible and modular design for easy expansion and scenario adaptation	Equipped with three independently steerable in-wheel motors and adopts a linear mechanism	(Quaglia et al., 2024)
		An unmanned transport vehicle suitable for greenhouse environments	Provide stable walking capability, automatic unloading, and autonomous operation	(Guan et al., 2025)
		Automated, maintainable, and eco-friendly mobile agricultural robot	Photovoltaic charging stations charge on board batteries	(Majdoubi and Masmoudi, 2021)
	Biological characteristics	Simulating greenhouse internal climate with a lumped capacitance thermal model	Evaluation of energy utilization and microclimate prediction	(Nauta et al., 2024)
		Light source optimization significantly improves Chinese cabbage growth.	Wireless sensors and control networks maintain plant factory growth conditions.	(Duc et al., 2021)
		A soil aeration system	Improving the soil environment through aerated cultivation	(Wang et al., 2025)
		A combination of high planting density and plant factories	Effects of cultivation facilities on the growth of tomato seedlings	(Zhang et al., 2025a)

For environmental perception and target recognition, the robot scans the operating environment with its visual system to establish a dynamic spatial coordinate system and uses image processing algorithms to identify the position, color, shape, and ripeness of mature fruits for screening harvestable targets. For path planning and navigation, based on perceived data, the robot employs obstacle avoidance algorithms and navigation technologies to generate an optimal movement path, ensuring the robotic arm safely and efficiently approaches target fruits while adapting to complex terrain and obstacles. For execution and grasping, the robotic arm moves along the planned trajectory, and the end effector completes grasping, separation, and placement actions to achieve precise fruit picking while preventing damage to adjacent fruits or crops. For biological characteristics, the system is designed to adapt to the unique biological traits of the target plant, including soft tissue texture, irregular surface morphology, and varying ripening periods, to minimize mechanical damage during the picking process.

### 4.1 Application of open field environments

Open field flower cultivation areas are fully exposed to natural conditions, where illumination intensity can plummet from a midday peak of 1500 µmol/m²/s to below 50 µmol/m²/s on overcast days or at dawn and dusk. Coupled with minute-level abrupt changes caused by cloud movement, vision systems struggle to maintain stable imaging quality across such a wide dynamic range. Strong light causes overexposure in petal highlight regions with loss of texture information, while low light reduces the contrast between target and background; both extremes can reduce target detection recall by 20%-30%. In contrast, greenhouse environments, regulated by shading nets and diffusing glass, typically restrict illumination to the relatively narrow range of 200-800 µmol/m²/s, allowing vision systems to optimize fixed exposure parameters and achieve significantly improved recognition stability.

Wind induced target motion constitutes another non-negligible disturbance in open fields. A wind speed of 3 m/s is sufficient to cause flower branches to sway with an amplitude of 50–100 mm at a frequency of 1–2 Hz; when wind speed reaches 8 m/s, the amplitude can exceed 200 mm with accompanying low frequency torsion. Under such dynamic conditions, static single-frame-based localization methods suffer from errors amplified to ±30–50 mm, far exceeding the typical ±10 mm tolerance range of end effectors, leading to a sharp increase in grasping failure rates. In facility environments, wind speed is typically controlled below 0.5 m/s, flowers remain essentially stationary, localization errors can be stabilized within ±5 mm, and visual servo control becomes substantially easier to implement.

The execution system faces equally severe challenges in open fields. Diurnal temperature variations of 15-20 °C and relative humidity fluctuations of 40%-95% not only alter the moisture content and stiffness of flower stem xylem, but also affect the measurement accuracy of force/torque sensors. Moreover, the undulating terrain and interlaced furrows require mobile platforms to possess a climbing capability of ≥15° and a ground clearance of ≥100 mm to ensure trafficability. The superposition of these multiple factors makes the system design complexity of open field flower picking robots far higher than that of facility environments.

### 4.2 Application of facility controllable environments

Facility environments such as greenhouses, plastic tunnels, and plant factories offer the core advantages of partially controllable environmental parameters, regularized planting layouts, and relatively constrained spatial scales. These characteristics both facilitate robot operations and give rise to new technical constraints.

Planting furrow spacing in facilities is typically 600–800 mm with furrow heights of 200–300 mm, requiring the robot body width to be controlled within 500 mm for flexible traversal between furrows, while the robotic arm’s workspace must cover the furrow width and plant height. Ouyang (2024) innovatively integrated QFD and TRIZ methodologies in the design of a harvesting and transport vehicle for fresh cut roses in greenhouses, with the chassis width precisely designed to pass through the gaps of various pipelines and move flexibly inside the sheds, thereby making the harvesting and packaging of fresh cut roses more systematic. Meanwhile, dense obstacles such as trellises, support wires, and irrigation pipes impose additional demands on local obstacle avoidance and precise end effector maneuvering capabilities. At the perception level, although facility environments maintain illumination within a relatively narrow range, the canopy closure in high density planting can reach 0.7-0.9, resulting in occlusion rates of flowers by upper leaves of 40%-70%, significantly higher than the 20%-40% observed in open fields, making it difficult for single view vision systems to fully capture the spatial pose of flowers and requiring the integration of multiple view imaging or active depth sensing to enhance localization robustness. At the execution level, facility grown flowers generally possess higher economic added value; taking fresh cut roses as an example, petal abrasion exceeding 2 mm² or an uneven stem cut may result in downgrading of the entire flower, compelling the end effector to maintain force control precision within ±1 N, achieve cut surface smoothness better than 0.5 mm, and incorporate compliant materials on gripping surfaces to limit contact pressure below 0.2 MPa. These constraints collectively determine that flower picking robots in facility environments must achieve coordinated optimization of trafficability, perception robustness, and operational precision within confined spaces.

### 4.3 Comparison of field type and facility type robots

**Figure 10:**
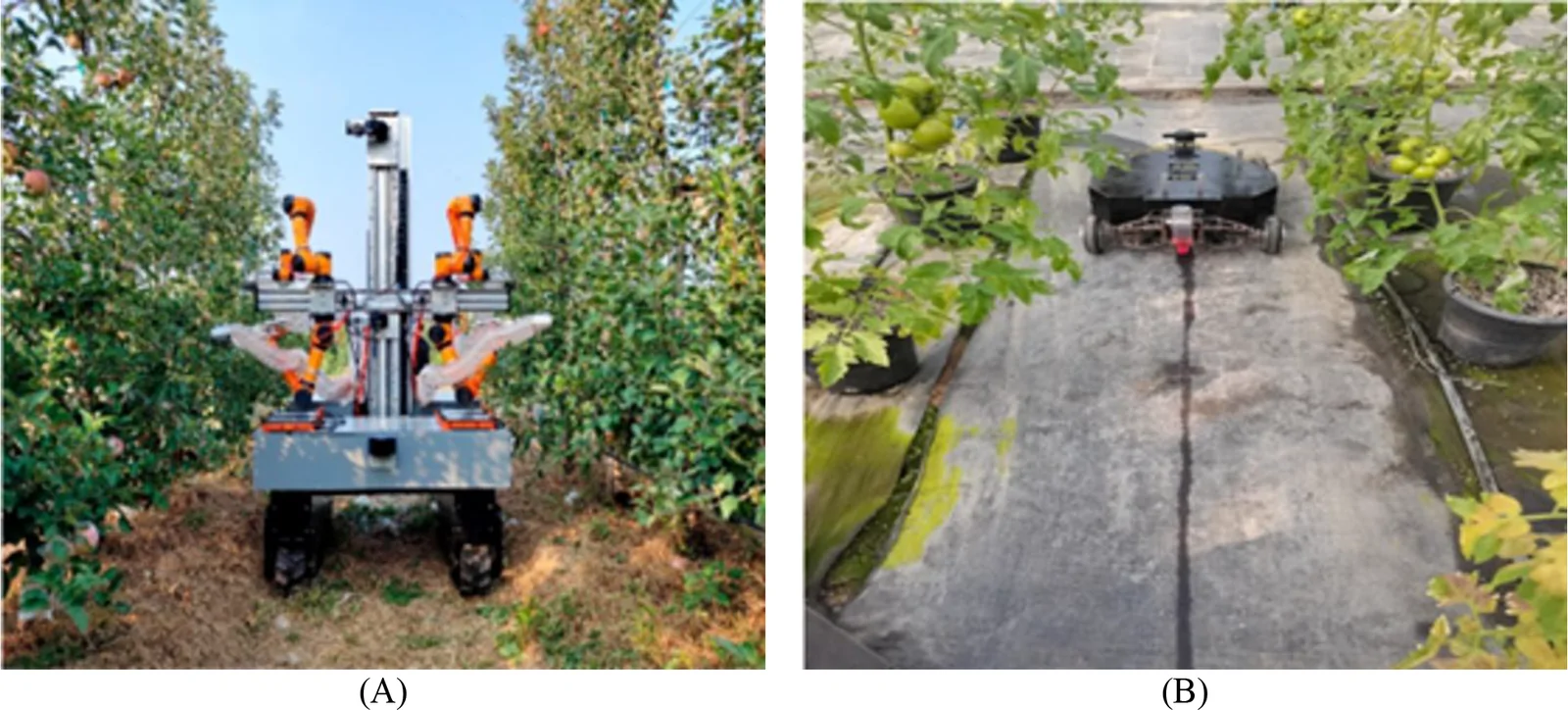
Different types of field robots and facility robots. **(A)** Four-arm field robot; **(B)** Picking facility robot chassis.

Field robots and facility robots are distinguished primarily by their working environments, which dictate fundamentally different parameter configurations, operational priorities, and design precautions across all key technical dimensions, as illustrated in **Figure 10**. **Figure 10A** shows a four-arm field robot designed for large scale outdoor operations, while **Figure 10B** depicts a facility robot chassis engineered for precise maneuvering within greenhouse environments.

Facility robots, on the other hand, focus on precise operations within a space. Their core technologies lie in high precision perception and motion control, dexterous end effector design, and flexibility and stability in narrow spaces, as illustrated in **Figure 10B**. The core goal is to achieve high quality and highly consistent picking in a controlled but complex limited space, meeting the needs of high end crops such as roses and orchids.

Field robots operate outdoors in complex, unstructured conditions where illumination fluctuates diurnally and seasonally, weather changes unpredictably, and wind induces continuous plant motion. The biological targets are genetically diverse cultivars with inconsistent growth habits, and the unstructured field offers no physical support or reference markers. Facility robots, by contrast, operate within controlled frameworks such as greenhouses, where environmental parameters are partially regulable, planting rows are geometrically regular, and flower varieties are often selected for uniform growth and predictable phenology. The constraint space in facilities is narrower and more stable, enabling tighter coupling between perception and action.

In terms of vision systems, field robots require robustness to changing natural illumination, shadow patterns, and dynamic backgrounds caused by wind and camera motion. Perception must handle occlusion by dense foliage, distinguish flowers from visually similar organs, and generalize across varieties using minimal retraining data. Facility robots benefit from controlled lighting, consistent backgrounds, and fixed camera to target geometries. Their perception systems can exploit prior knowledge of row structure and plant spacing, achieving higher detection rates and more precise localization with lower computational overhead, though they must still handle high density planting and frequent occlusions within a much more predictable visual domain.

Regarding robotic arm configurations and motion planning, field robots typically employ longer reach, higher payload arms mounted on mobile platforms to accommodate uneven terrain and widely spaced plants. Motion planning must cope with large scale workspace uncertainties, requiring real-time replanning in response to platform sway, unexpected obstacles, and target displacement, with collision avoidance complicated by the lack of a complete environmental model. Facility robots use shorter, lighter, and faster arms with well-defined workspaces relative to fixed planting beds. Motion planning can be pre-optimized for repetitive pick and place cycles with high speed trajectories and minimal clearance tolerances, relying on accurate prior maps of the greenhouse layout for efficient offline or hybrid online offline planning.

End effector design embodies the differing priorities between robustness in the field and precision in the facility. Field robots demand mechanically robust, weather resistant end effectors tolerant to significant positional errors, accommodating wide variability in stem diameter, flower orientation, and tissue stiffness across seasons and cultivars while functioning reliably despite dirt, moisture, and temperature extremes, with actuation simple enough for field maintenance. Facility robots can employ more delicate, high precision end effectors with integrated tactile and force sensing, fine tuned to specific flower varieties grown under controlled conditions. The emphasis in facilities is on minimizing cycle time while achieving zero damage rates, often using soft, compliant materials and sensor rich designs that require careful calibration and clean environments.

The mobile platforms of the two categories reflect fundamentally different terrains and spatial layouts. Field robots require rugged platforms capable of traversing uneven, soft, or slippery terrain, climbing slopes, and navigating around obstacles such as irrigation pipes and support posts. Chassis design must incorporate active or passive suspension systems for attitude stabilization, while localization relies on GPS/RTK, inertial navigation, and visual odometry fused in real time, with power systems supporting extended field operation under limited recharging infrastructure. Facility robots use wheeled or rail guided platforms operating on level, prepared surfaces. Localization is simplified by ceiling mounted markers, RFID, or visual landmarks, and mobility emphasizes smooth, repeatable path following, precise docking at planting rows, and coordinated multiple robot scheduling within confined spaces, with power supplied via tether or frequent automated recharging stations.

In essence, field robots are designed to cope with the unknown, whereas facility robots are optimized for peak performance within known boundaries. Together, these two categories of robots are driving a profound transformation in agricultural production from machine assisted to autonomous and intelligent operations.

## 5 Technical challenges and outlook

**Figure 11:**
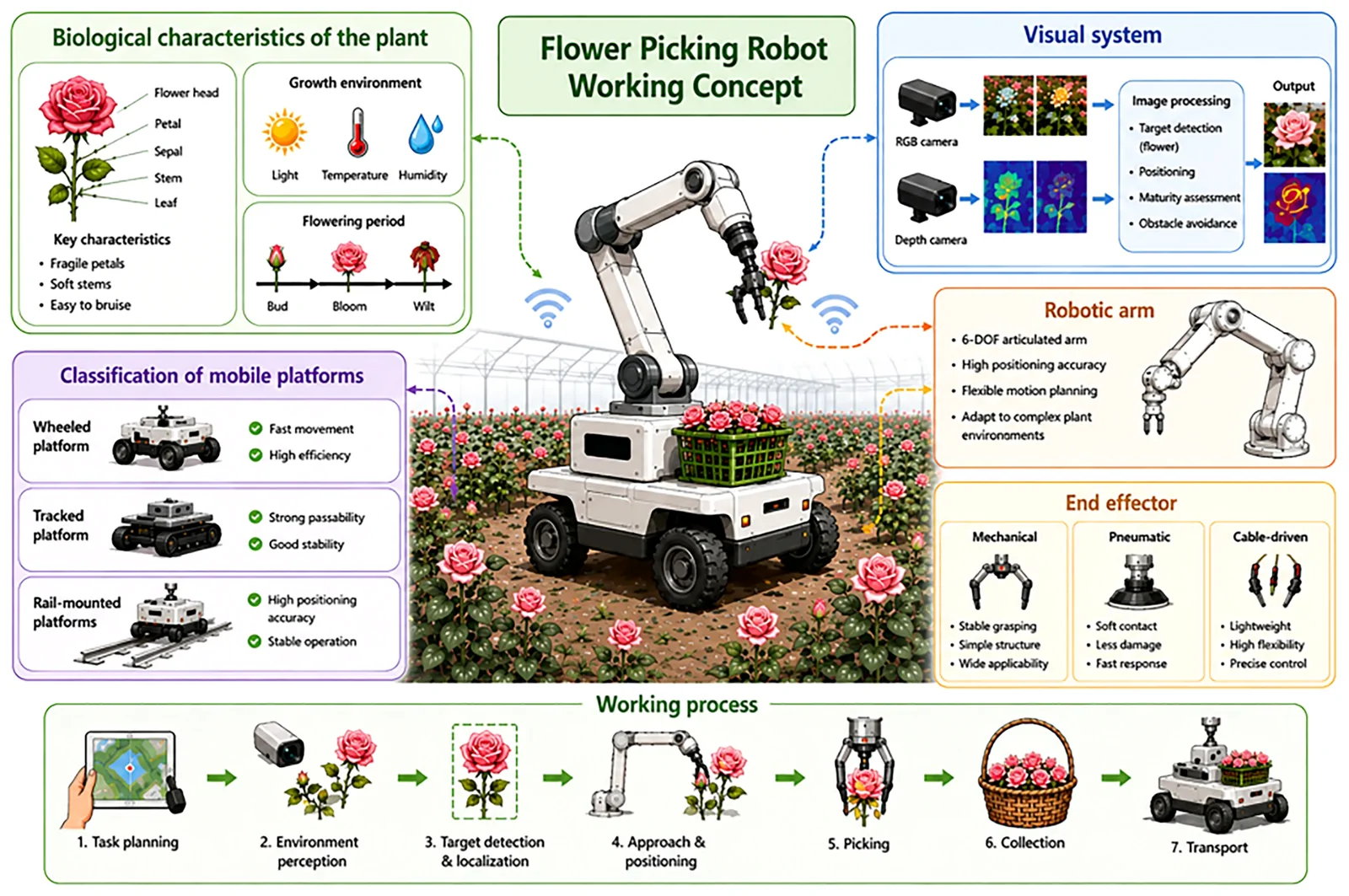
Flower picking robot working concept.

The evolution of flower picking robot marks a decisive shift in agricultural automation, moving from standardized field operations toward high value, precision sensitive tasks. Despite substantial progress across perception, actuation, and decision-making subsystems, current systems remain constrained by three interconnected bottlenecks that together form a compounding chain of difficulty, namely that a robot cannot manipulate what it cannot characterize, and it cannot characterize what it cannot reliably perceive. These three bottlenecks are not independent; they form a pipeline in which failure at any stage cascades forward. The complete operational workflow of a typical flower picking robot, encompassing the full chain from environmental perception and target recognition to trajectory planning and non-destructive execution, is illustrated in **Figure 11**. This architecture, while functionally coherent, exposes the very interdependencies that give rise to the three bottlenecks discussed above. Addressing them, therefore, requires a coordinated research agenda organized around three progressive frontiers, including digitizing the environment, cognizing the object, and dexterously realizing the action.

### 5.1 Multiple modal perception and target characterization

Visual cues including color, shape, and size are insufficient for parameterizing non-destructive picking. Critical properties such as tissue compliance, surface friction, internal hydration, and bruise susceptibility lack reliable visual proxies. Fusing high resolution tactile arrays, thermal imaging, near infrared spectroscopy, and volatile organic compound sensors offers a path toward non-destructive inspection. Thermal signatures reveal transpiration patterns indicative of petal freshness, short wave NIR reflectance correlates with water status and sugar content, and contact mode tactile imaging maps local stiffness variations at submillimeter resolution before grasp commitment.

The key challenge lies in spatiotemporal alignment of multiple modal data. Visual frames arrive at 30 to 60 Hz, tactile readings at 100 to 1000 Hz, and spectral measurements at sub-Hertz rates; fusing these asynchronous streams remains an open problem. Another challenge is varietal diversity, making dedicated models for each target infeasible. Foundation models pretrained on large scale plant phenotyping corpora and fine tuned with minimal examples enable recognition of novel varieties from few labeled instances, allowing deployment across thousands of commercial varieties without prohibitive data collection costs.

### 5.2 Bionic and soft robotic end effectors

Even with accurate environmental models and target characterization, the robot must plan collision-free trajectories through cluttered canopies, establish compliant contact, modulate force at the millinewton level, and complete separation within viable cycle times (Li-wen et al., 2023). Embedding digital twins into high-fidelity physics simulators enables reinforcement learning agents to perform complex maneuvers including branch avoidance, leaf displacement, and end effector orientation without costly trial-and-error on real flowers (Kim et al., 2023). Conventional rigid grippers operate on a single force-displacement profile that cannot satisfy both gentleness during approach and rigidity during cutting. Even minor vibrations can bruise or abrade delicate petals (Lv et al., 2022). Bio-inspired stiffness configurable designs, such as pneumatic soft fingers that wrap compliantly and stiffen via granular jamming or magnetorheological fluids, decouple these conflicting requirements. Chassis self-leveling through multiple link suspensions or wheel-leg mechanisms counteracts terrain-induced roll, pitch, and heave, transforming unpredictable field surfaces into virtual level ground. High-bandwidth visuotactile servoing with gel-based optical tactile sensors and force-position hybrid control detects incipient slip and modulates grasp force in real time, which is essential for high-value varieties where a single bruised petal renders the flower unsalable.

### 5.3 Multiple robot collaborative systems

These three frontiers form an interdependent agenda. Progress in isolation yields diminishing returns, the decisive advance comes from integration into a unified architecture where each stage feeds the next and adapts to feedback from the last (Wu et al., 2022).

In large scale applications, multiple robot collaborative systems enhance efficiency through task allocation, path coordination, and information sharing. Given residual uncertainty in target state estimation, robots must address collaborative planning in cluttered canopies, with wind requiring real-time compensation, terrain unevenness demanding self-leveling, and morphology and mechanics determining workspace and strategy. Multiple robot systems distribute these complexities across heterogeneous units, for example with one robot handling global perception and mapping while others execute picking based on the shared map. Mobile collaborative platforms allow simultaneous operation, increasing harvesting density per unit time. Current research remains at the laboratory prototype stage; large scale system integration and validation under real greenhouse conditions remain to be addressed.

## 6 Conclusion

Flower picking robots are currently at a critical stage of transitioning from laboratory scale principle validation to field trials in specific scenarios. From a system architecture perspective, this paper has provided a systematic review of recent research progress in flower picking robots, focusing on three core technologies, namely environmental perception and target recognition, motion control and path planning, and compliant non-destructive end effector design.

In terms of environmental perception, deep learning-based visual detection algorithms have achieved promising results under controlled conditions. However, in open field environments, severe illumination fluctuations and wind induced target motion significantly reduce detection recall and localization accuracy, indicating that the robustness of existing algorithms still falls short of practical operational requirements. Regarding motion planning, improved path planning algorithms have demonstrated effective obstacle avoidance capabilities in simulations and limited field trials, yet the real-time performance and adaptability to dynamic disturbances in the dense, unstructured canopy environments characteristic of flower cultivation require further enhancement. For end effectors, pneumatically driven and cable driven soft grippers have validated the feasibility of non-destructive grasping under laboratory conditions, but existing designs lack adaptive capabilities to accommodate the significant variations in geometry and mechanical properties across different flower varieties, limiting their cross variety applicability.

In terms of application environments, facility environments generally achieve higher picking success rates due to controllable illumination and minimal wind induced disturbances, yet impose more stringent requirements on end effector force control precision and cut quality. Open field environments, in contrast, face multiple coupled disturbances including illumination, wind, and terrain variations, resulting in significantly higher system design complexity. Existing studies are largely single technology validations conducted for specific varieties under specific conditions, and have not yet formed universal solutions applicable across multiple varieties and scenarios.

In summary, flower picking robots remain considerably distant from large scale commercial deployment. At the current technological level, they have demonstrated application potential in structured greenhouse environments for a limited number of high value flower varieties, but still face fundamental challenges in open field environments and multiple variety adaptability. Future research should focus on the following key directions, including multiple modal sensor fusion and robust perception adaptable to dynamic environments, adaptive compliant end effector design accommodating flower diversity, and system level integration optimization of the full perception, planning and execution pipeline.
